# The Official Languages at the International Medical Congress at Moscow in 1897

**Published:** 1896-06

**Authors:** 


### The Official Languages at the International Medical Congress at Moscow in 1897.

Yielding to the universal protest, the Committee of Management have announced that English and German will also be accepted as official languages, with French and Russian.

